# Livestock-associated methicillin-resistant *Staphylococcus aureus* responsible for human colonization and infection in an area of Italy with high density of pig farming

**DOI:** 10.1186/1471-2334-13-258

**Published:** 2013-06-03

**Authors:** Monica Monaco, Palmino Pedroni, Andrea Sanchini, Annalisa Bonomini, Annamaria Indelicato, Annalisa Pantosti

**Affiliations:** 1 Department of Infectious, Parasitic and Immuno-mediated Diseases, Istituto Superiore di Sanità, viale Regina Elena, 299, Rome, Italy; 2 Presidio Ospedaliero Manerbio-Leno, Azienda Ospedaliera Desenzano del Garda, Lungomella Valsecchi, Brescia, Italy; 3 Present address: Robert Koch-Institut (ZBS 1), Nordufer 20 D-13353 Berlin-Wedding, Berlin, Germany; 4 Present address: European Public Health Microbiology Training Programme (EUPHEM), European Centre for Disease Prevention and Control (ECDC), Tomtebodavägen 11a, 171 83, Stockholm, Sweden

**Keywords:** Livestock-associated (LA)-MRSA, Colonization, Community-acquired infections, Pigs, ST398

## Abstract

**Background:**

Livestock-Associated MRSA (LA-MRSA) belonging to ST398 lineage, common among pigs and other animals, emerged in Central and Northern Europe, becoming a new risk factor for MRSA among farm workers. Strains belonging to ST398 can be responsible for human colonization and infection, mainly in areas with high livestock-farming. The aim of this study was to investigate the occurrence of livestock-associated methicillin-resistant *Staphylococcus aureus* (LA-MRSA) human colonization and infections in an area of the Lombardy Region (Italy), the Italian region with the highest density of pig farming.

**Methods:**

In the period March-April 2010, 879 nasal swabs were taken from subjects at admission to a local hospital serving an area of the Lombardy Region devoted to agriculture and farming. In the period March 2010-February 2011, all MRSA strains from community-acquired infection (CAI) observed in the same hospital, were collected. Molecular characterization of the isolates included SCC*mec* typing, *spa* typing and multilocus sequence typing (MLST).

**Results:**

Out of 879 nasal swabs examined, 9 (1%) yielded MRSA. Five strains were assigned to sequence type (ST)398 (*spa* t899, 3 isolates; t108 and t2922, 1 isolate each) and were therefore categorized as LA-MRSA. The other 4 isolates were likely of hospital origin. No strains were positive for Panton-Valentine Leukocidin genes. Twenty MRSA isolates were detected from CAI, 17 were from skin and soft-tissue infections and 3 from other infections. An MRSA isolate from otitis externa was t899/ST398 and PVL-negative, hence categorized as LA-MRSA. Four isolates were assigned to t127/ST1. Eight strains were PVL-positive community acquired (CA)-MRSA and belonged to different clones, the most frequent being ST8.

**Conclusions:**

In an area of Italy with high density of pig farming, LA-MRSA is able to colonize the population and rarely to produce infections. Typical CA-MRSA is more common than LA-MRSA among CAI.

## Background

Methicillin-resistant *Staphylococcus aureus* (MRSA) emerged in hospital fifty years ago and swiftly became one of the most important hospital-associated (HA) pathogens being responsible for serious infections such as pneumonia and sepsis [1], and commonly characterized by a multi-drug resistant phenotype. Distinct lineages of HA-MRSA emerged in the years and became established with different success in different geographical locations [2].

Starting from the last decade, MRSA epidemiology largely changed, due to the emergence of new MRSA lineages, responsible for infections occurring in the community among patients without known risks factors for the acquisition of MRSA and without previous hospital contacts [3]. These strains, defined community acquired (CA)-MRSA, are mainly responsible for skin and soft-tissue infections (SSTI), although deep-seated infections such as necrotizing pneumonia, sepsis and meningitis, have also been reported [3,4]. CA-MRSA is characterized by the presence of the Panton-Valentine Leukocidin (PVL), a toxin that causes polymorphonuclear lysis and tissue necrosis [5] although its contribution to disease is still debated [6,7].

More recently, the emergence of an MRSA clone colonizing pigs and, more rarely, other farm animals (cattle and poultry) have been reported in Europe [8-10]. These strains, designated livestock-associated (LA)-MRSA and belonging to sequence type (ST)398, were mostly found in countries with high density of pig farming such as the Netherlands, Denmark and Germany [11,12]. These strains were able to colonize persons working in close contact with pigs, such as farmers and veterinarians. In Netherland and in Germany, 26.5% and 24% of pig farmers were colonized by LA-MRSA, respectively [12,13]. Wulf *et al.* found that 12.5% of veterinarians attending an international conference were colonized by MRSA of which 92.5% were LA-MRSA [14]. Farmers’ family members, who are not in direct contact with pigs, were colonized at a lower frequency than the farm workers, indicating that inter-human transmission may occur [15].

Since LA-MRSA was found in dust from pig holdings [16], the environment could represent a vehicle for the transmission of LA-MRSA strains from animals to humans, allowing colonization of subjects who do not work directly with animals.

Infections due to LA-MRSA have been reported occasionally in farm workers and their family members [10]. In the Netherlands and Denmark where the percentage of HA-MRSA is very low (<1%), LA-MRSA strains represent an important reservoir for human MRSA infections [10,11]. In the Netherland, by the end of 2008, LA-MRSA ST398 accounted for 42% of all new detected MRSA [12].

In Italy, LA-MRSA ST398 prevalence in farm workers is unknown. In the study previously cited, MRSA carriage in Italian veterinarians was found to be 54% [14]. Two cases of serious infections due to LA-MRSA were described in Italian farmers in the Lombardy region: a man working on a pig farm who developed cellulitis and pyomiositis of the buttock and a man working on a dairy farm who developed necrotizing fasciitis of the neck [17,18].

Other MRSA lineages different from ST398 were found in a European survey on the prevalence of MRSA in pig holdings. One lineage, characterized by *spa* type t127 and belonging to ST1 was predominantly reported from Italy [16], as shown also by other studies [19]. Franco *et al.* recently demonstrated that t127/ST1 isolates can be assigned to two genetically different clusters (porcine and human) and hypothesized that t127/ST1 strains could represent another lineage of LA-MRSA [20].

This study was undertaken to investigate the prevalence of LA-MRSA colonization and the occurrence of LA-MRSA infections in subjects living in an area of the Lombardy region, where livestock farming is a prominent activity.

## Methods

### Setting of the study and source of isolates

**Figure 1:**
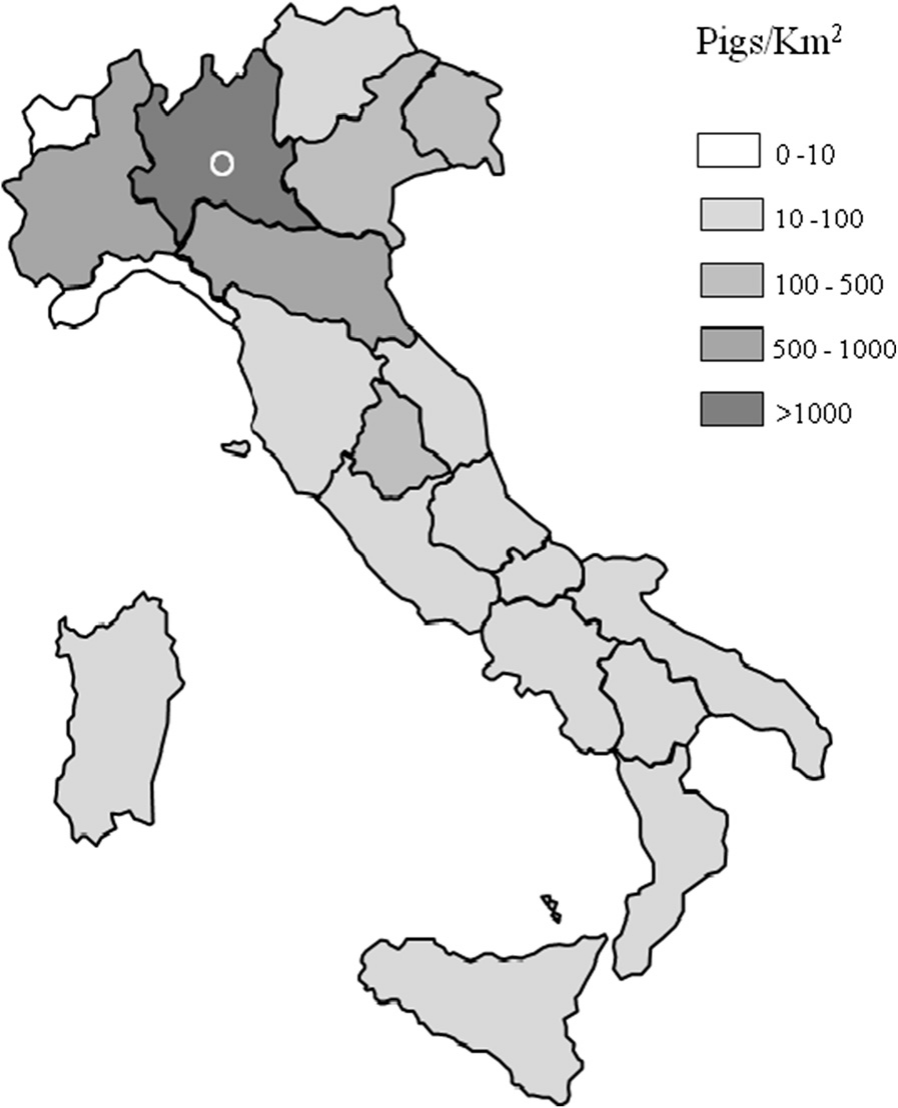
**Density of breeding and fattening pigs in Italy.** Adapted from [21]. The open circle indicates the area in the Lombardy Region where the study was performed.

Two prospective studies were performed: a study on MRSA colonization in patients at hospital admission and a study on MRSA in community-acquired infections (CAI). Both studies were conducted at the Manerbio Hospital, a local hospital with 250 beds, serving a population of approximately 120.000 inhabitants. This area was chosen for the studies because a case of LA-MRSA infection was detected in 2008 [18], and because livestock farming is one of the main economical activities of this area, located in Lombardy, the region with the highest pig farming density in Italy (Figure 1) [21].

For the colonization study, in the period March-April 2010, all subjects presenting to the hospital for pre-admission laboratory tests before planned surgery, were subjected to a nasal swab for MRSA screening. Swabs were plated onto Mannitol Salt agar and Brilliance MRSA agar plates (Thermo Fisher Scientific, Milan, Italy). No enrichment culture was performed. The isolates were identified using an automated system (Vitek2, bioMérieux, Marcy-l’Etoile, France) at the Manerbio Hospital laboratory. Interpretative criteria were those suggested by the Clinical and Laboratory Standards Institute (CLSI) [22].

The study on CAI was conducted from March 2010 to February 2011. The definition of CAI included infections in outpatients and in hospitalized patients, within 48 h from hospital admission, who had not been hospitalized in the previous 3 months. MRSA strains originating from CAI were collected and further characterized. Only the first MRSA isolate per patient was studied.

Nasal screening was part of the pre-admission routine tests for surgical patients at the hospital involved in the study. Patients gave informed consent to the surgical procedure. Clinical data and samples (bacterial isolates) were anonymised before being used for the study. The study was exempt from Ethics Committee approval according to local and national regulations.

### Antimicrobial susceptibility testing

In vitro susceptibility tests were obtained by an automated system (Vitek2, bioMérieux, Marcy-l’Etoile, France). The antimicrobial agents tested included: oxacillin, gentamicin, levofloxacin, erythromycin, clindamycin, tetracycline, rifampicin, vancomycin, teicoplanin, tigecycline, linezolid, trimethoprim-sulfamethoxazole, fusidic acid and mupirocin. Interpretative criteria were those suggested by the CLSI [22].

### Molecular characterization of the isolates

Genomic DNA of MRSA isolates was extracted by the QIAamp DNA Mini Kit (QIAGEN, Hilden, Germany) and used as a template for polymerase chain reaction (PCR) amplification. *S. aureus* species and methicillin resistance status were confirmed by a duplex PCR targeting the genes *nuc* and *mec.* This PCR assay was developed in our laboratory by using primers published by Brakstad O.G *et al.*, and Murakamy K., *et al.*[23,24]. The detection of the Panton-Valentine leukocidin (PVL) and of the arginine catabolic mobile element (ACME)-specific *arc*A gene were performed as previously described [25]. The *sak* gene, encoding the plasminogen activator staphylokinase, was investigated on t127 strains by using published primers [26].

To determine the Staphylococcal chromosomal cassette *mec* (SCC*mec*) type, a combination of different multiplex PCR assays, discriminating the *mec* complex and the *ccr* genes complex, were applied by using protocols previously described [25]. Subtyping of the SCC*mec* type IV was obtained applying the method described by Milheiriço C., *et al.*[27].

Amplification, sequencing and analysis of the polymorphic region of the protein A gene (*spa* typing) was performed according to the standard protocol of Harmsen *et al.*[28]. MLST was performed on selected strains as described elsewhere [29]. The allelic profiles obtained were compared to those present in the MLST database.

## Results

In the colonization study, 9 out of 879 patients (1%) subjected to nasal screening prior to hospitalization were positive for MRSA. The age of patients carrying MRSA ranged from 17 to 71 years (median age 58); 4 patients were females and 5 were males. Out of the 9 patients colonized, 5 (56%) carried LA-MRSA ST398. The overall LA-MRSA colonization rate was 0.56%.

The characteristics of the MRSA isolates are shown in Table 1. The 5 LA-MRSA strains were t899/SCC*mec* type IVa (2 isolates), t899/SCC*mec* IVc (1 isolate), t108/SCC*mec* type V (1 isolate) and t2922/SCC*mec* type V (1 isolate). The other isolates obtained from carriage belonged ST8 (3 isolates) and ST5 (1 isolate). None of the colonizing strains was positive for PVL genes.

**Table 1:** Clinical informations, genotypic and phenotypic characteristics of MRSA responsible for human colonization and infections

**Isolate**	**Patient gender**	**Patient age (years)**	**Type of infection**	**SCC*mec *type**	***spa *type**	**ST**	**CC**	**PVL**	**ACME**	**Resistance pattern to non beta-lactam antibiotics**^**§**^
Colonization										
Sau93	M	67	-	IVc	t899	398	398	-	-	ERY, CLI, SXT, TET
Sau95	F	77	-	IVa	t899	398	398	-	-	ERY, CLI, TET
Sau98	F	58	-	IVa	t899	398	398	-	-	TET
Sau91	M	40	-	V	t108	398	398	-	-	LVX ^#^, TET
Sau99	M	20	-	V	t2922	398	398	-	-	ERY, CLI, SXT, TET
Sau96	F	61	-	IVc	t008	8	8	-	-	LVX, TET
Sau97	M	66	-	IVc	t008	8	8	-	-	GN, LVX
Sau92	M	71	-	IVc	t008	8	8	-	-	LVX
Sau94	F	17	-	V	t688	5	5	-	-	ERY, CLI*, TET
Infections										
Sau150	M	18	SSTI	IVa	t008	8	8	+	+	LVX, ERY
Sau116	F	71	SSTI	IVa	t008	8	8	+	+	LVX, ERY
Sau161	F	27	SSTI	IVa	t008	8	8	+	+	LVX, ERY
Sau85	F	46	SSTI	IVa	t121	8	8	+	-	LVX, ERY
Sau86	F	35	SSTI	IVc	t005	22	22	+	-	ERY, CLI*
Sau149	M	0.8	SSTI	IVc	t044	80	80	+	-	FUS, TET
Sau151	F	2	SSTI	V	t127	1	1	+	-	GN, FUS, TET
Sau119	M	38	SSTI	V	t7445	772	1	+	-	GN, ERY, CLI* SXT,
Sau118	F	53	SSTI	IVc	t008	8	8	-	-	LVX ^#^,
Sau73	F	70	SSTI	IVc	t008	8	8	-	-	LVX ^#^, ERY, CLI*
Sau159	F	85	SSTI	IVc	t008	8	8	-	-	LVX
Sau163	F	53	SSTI	IVc	t008	8	8	-	-	GN, LVX, RIF
Sau84	M	67	SSTI	IVc	t008	8	8	-	-	LVX, TET
Sau121	F	83	BSI	IVc	t008	8	8	-	-	LVX
Sau82	F	84	SSTI	IVa	t127	1	1	-	-	LVX, ERY, CLI*
Sau83	F	9	SSTI	IVa	t127	1	1	-	-	ERY, CLI*, TET
Sau162	F	61	OE	IVa	t127	1	1	-	-	GN, ERY, CLI*, TET
Sau120	M	77	SSTI	IVh	t515	22	22	-	-	LVX ^#^
Sau117	M	79	SSTI	I	t041	228	5	-	-	GN, LVX, ERY, CLI, RIF^#^
Sau74	M	68	OE	IVc	t899	398	398	-	-	CLI, SXT, TET

SCC*mec* staphylococcal cassette chromosome *mec*, ST sequence type, CC clonal complex, PVL Panton-Valentine Leukocidin, ACME arginine catabolic mobile element.

^§^ GEN gentamicin, ERY erythromycin, CLI clindamycin, LVX levofloxacin, FUS fusidic acid, TET tetracycline, SXT co-trimoxazole, RIF *rifampicin*, * inducible clindamycin resistance; ^#^ intermediate resistance.

SSTI skin and soft tissue infection, BSI bloodstream infection, *OE* otitis externa.

In the infection study, 20 CAI due to MRSA were detected in the span of one year. Thirteen patients were females and 7 were males. The age of patients ranged from 8 months to 85 years (median age 53). Out of the 20 isolates, 17 were obtained from SSTI, 2 from otitis externa and 1 from bloodstream infection. One of the isolates from otitis externa was t899/ST398/SCC*mec* IVc and was categorized as LA-MRSA. Eight strains were positive for PVL and were considered typical CA-MRSA. Of these, 3 were t008/ST8/SCC*mec* IVa and ACME-positive and therefore most likely corresponding to the USA300 clone. Another strain was t044/ST80/SCC*mec* IVc and belonged to the European clone, while 3 other strains belonged to other lineages (ST22, ST772 and ST1).

ST8 and ST1 were found among both PVL-positive and PVL-negative isolates. PVL-negative ST8 were negative for ACME, too. All ST1 isolates (4 strains), belonged to *spa* type t127 and harboured the *sak* gene; of these one carried also PVL. The PVL-positive t127 isolate carried SCC*mec* V while the 3 PVL-negative t127 isolates carried SCC*mec* IVa.

All the isolates, both from colonization and infections, were fully susceptible to vancomycin, teicoplanin, linezolid, tigecycline and mupirocin. All ST398 isolates were resistant to tetracycline. Resistance profiles to other non-beta-lactam antibiotics are shown in Table 1.

## Discussion

In the present study we examined MRSA strains obtained from nasal screening of subjects prior to hospital admission and MRSA strains from CAI in an Italian Region, Lombardy, with the highest density of pig farming in Italy (48.2% of all the pigs reared in the country) [21]. In particular, in the area where the study was conducted, a serious case of LA-MRSA infection was identified in 2008 [18].

Although the number of MRSA isolates obtained in this study was small, two different pictures emerged clearly: LA-MRSA, belonging to ST398, was prevalent (56%) among the admission screening MRSA isolates, while, among the infections typical PVL-positive CA-MRSA predominate and only one strain belonging to ST398 was found. The percentage of LA-MRSA out of all MRSA colonizing strains is similar to that found in patients at admission to hospital in a region with high density of animal farming in Germany [30].

Unfortunately we were unable to obtain precise information about the working activity of the colonized subjects or of the infected patients, therefore we do not know if any of them worked or lived in close contact with pigs or other farm animals. The overall percentage of ST398 colonization in our study (0.56%) is in the range (from <0.01 to 1.2%) described in the Netherlands and Germany, regarding non-exposed healthy population [12,13].

Our study confirms that the presence of LA-MRSA in human infections is rare. LA-MRSA isolates have been reported to sporadically cause mild or serious infections and a hospital outbreak [17,18,31,32]. According to data reported by Van Cleef *et al.*, MRSA ST398 clone contributes only to a small fraction (<2%) of all MRSA isolates in humans [9]. In a framework of an European survey conducted in 2011 on invasive infections caused by *S. aureus* predominant clones, we found that in Italy 1 out of 97 MRSA (1.5%) strains belonged to a *spa* type indicative of ST398 (unpublished data), while in a previous survey carried out in 2006 no ST398 was found [2]. Therefore the ability of LA-MRSA to cause infections appears to be limited. This is probably due to the lack or rare occurrence of specific virulence factors, such as toxic shock syndrome toxic-1 (TSST-1) toxin and PVL, as shown by microarray and genomic studies [33,34]. In addition, LA-MRSA lacks the cluster of genes for human-specific immune evasion encoded by Saϕ3 bacteriophage, and therefore is less virulent in humans than other staphylococcal clones [34,35].

In our study on outpatient infections, a prominent role was played by typical CA-MRSA strains. The presence of a variety of CA-MRSA clones including USA300, has been observed, as already reported in other studies [4,25,36].

The recovery of isolates belonging to t127/ST1 is intriguing. t127/ST1 strains were recently isolated from pigs in Italy and the presence of a pig reservoir of this lineage has been hypothesized [20]. In the study by Franco *et al.*, the t127 strains of porcine origin carried SCC*mec* type V, while the t127 isolates of human origin harboured SCC*mec* type IVa and carried the *sak* gene, an immune-evasion factor present almost exclusively in human strains [20]. In our study three t127 strains carried SCC*mec* type IVa and the *sak* gene, indicating human origin. The only strain carrying SCC*mec* type V also harbored *sak* and PVL and therefore its porcine or human origin cannot be hypothesized.

Other clones causing infections are likely of hospital origin, such as the PVL-negative ST8 clone, resembling the “Lyon clone”, a well adapted hospital clone that emerged in France and spread in other European countries, including Italy [37,38]. The presence of HA-MRSA in infections in the community might be due to previous contact of the patients with health-care settings.

## Conclusion

Italy is one of the European countries with the highest MRSA rate according to data from the EARS-Net (around 40%) and with the highest density of pig-livestock farming [16]. This is the first study evaluating the prevalence of LA-MRSA in the general population, in carriage and infections, in our country.

The presence of LA-MRSA in the general population confirms the importance of the animal reservoir and indicate the need for preventive measures to control the extent of LA-MRSA spread.

## Abbreviations

MRSA: Methicillin-resistant *Staphylococcus aureus*; HA: Hospital-associated; HA-MRSA: Hospital-associated-MRSA; CA-MRSA: Community acquired-MRSA; PVL: Panton-Valentine leukocidin; LA-MRSA: Livestock-associated-MRSA; ST: Sequence type; CAI: Community-acquired infections; ACME: Arginine catabolic mobile element; SCCmec: Staphylococcal chromosomal cassette *mec*; MLST: Multilocus sequence typing; TSST-1: Toxic shock syndrome toxic-1; EARS-Net: European Antimicrobial Resistance Surveillance-Network; CC: Clonal complex; GEN: Gentamicin; ERY: Erythromycin; CLI: Clindamycin; LVX: Levofloxacin; FUS: Fusidic acid; TET: Tetracycline; SXT: Co-trimoxazole; RIF: Rifampicina; SSTI: Skin and soft tissue infection; BSI: Bloodstream infection; OE: Otitis externa.

## Competing interests

The authors declare that they have no competing interests.

## Authors’ contributions

MM participated in the design of the study, carried out the molecular analyses and drafted the manuscript. PP, AB and AI participated in the design of the study and provided isolates. AS helped to perform the molecular typing. AP conceived the design of the study and helped to draft the manuscript. All authors read and approved the final manuscript.

## Pre-publication history

The pre-publication history for this paper can be accessed here:

http://www.biomedcentral.com/1471-2334/13/258/prepub
